# A study on the diffusion model of new energy passenger vehicles with consideration of product value

**DOI:** 10.1371/journal.pone.0323316

**Published:** 2025-05-19

**Authors:** Zhongya Han, Dongyuan Zhao, Fengxia Sun, Huike Zhu

**Affiliations:** 1 Research Base of School of Economics and Management, Wenzhou University of Technology, Wenzhou, China; 2 Research Base of School of Economics and Management, Huaibei Normal University, Huaibei, China; 3 Research Base of Beijing Modern Manufacturing Development, School of Economics and Management, Beijing University of Technology, Beijing, China; 4 Research Base of School of Economics and Management, Anhui University of Finance and Economics, Anhui, China; Hamad Bin Khalifa University College of Science and Engineering, QATAR

## Abstract

Accurately forecasting new energy passenger vehicle sales is essential for developing effective marketing strategies and supporting government policies. Consumers purchase decisions for new energy passenger vehicles are primarily driven by product value, which is shaped by various product attributes that evolve through technological advancements. In this study, we develop a product value function for new energy vehicles based on the theory of value engineering. Then, the product value is integrated into the Bass model, and an Improved Bass Model based on Product Value (IBMPV) is proposed. The experiment results demonstrate that the IBMPV outperforms the Bass, Gompertz, Logistic and ARMAX models in terms of goodness of fit and predictive accuracy, making it more suitable for forecasting new energy passenger vehicle sales. The market potential for new energy passenger vehicles exhibits exponential growth as product value improves. Furthermore, we find that while enhanced product value increases the influence of external factors, it simultaneously reduces the influence of internal factors. This study provides a quantitative assessment of the role of product value on new energy passenger vehicle diffusion and presents a practical framework for sales forecasting.

## 1. Introduction

The automotive industry, a pivotal pillar of the national economy, faces increasing pressures from environmental concerns and the depletion of fossil fuels, driving the need for sustainable alternatives. The promotion of new energy vehicles, particularly in the passenger vehicle sector, has emerged as a key focus in China’s strategic emerging industries. This transition not only safeguards energy security but also mitigates environmental pollution and climate change, while driving the transformation and upgrading of the automotive industry [1,2]. Despite ongoing technological advancements and an increasingly supportive policy environment, the new energy vehicle industry still faces significant uncertainties, particularly in market demand and diffusion rate. According to data from China Association of Automobile Manufacturers, although the sales of new energy vehicles in China surged to 9.495 million units in 2023, with new energy passenger vehicles reaching 7.305 million, accounting for 76.9% of total new energy vehicle sales, the market remains highly dynamic and unpredictable. The immaturity of the new energy vehicle industry, coupled with rapid technological evolution and shifting consumer preferences, adds complexity to demand forecasting [3]. Accurate and reliable sales forecasting is essential for addressing these challenges, enabling enterprises to optimize investment and marketing strategies, enhancing supply chain efficiency, and supporting government policymakers in designing effective regulatory frameworks. Thus, developing robust forecasting models for new energy passenger vehicles is a pressing necessity for ensuring the stability and long-term success of the industry.

New energy passenger vehicles, classified as durable goods, are characterized by infrequent repeat purchases. Consumers typically engage in extensive information gathering and evaluation of product attributes—such as range, charging convenience, performance, and price—before making purchase decisions [4,5]. It is also worth noting that continuous technological advancements drive dynamic changes in these attributes, directly influencing product value, thus affecting consumer purchase behavior and new energy passenger vehicle sales [6]. Moreover, as an emerging industry, the new energy passenger vehicle market is constrained by limited historical data and market scale, posing challenges for traditional forecasting methods.

The Bass model, widely used for predicting durable goods sales, offers several advantages for new energy passenger vehicle sales forecasting. First, the Bass model exhibits good predictive performance for durable goods [7–9], and assumes that consumers do not make repeated purchases. Second, the Bass model can incorporate the influence of various factors, with adequate economic significance of the diffusion parameters, which can better explain the diffusion process and sales forecasting results of new energy passenger vehicles. Third, the Bass model requires a relatively small amount of historical data for fitting and forecasting. Therefore, the Bass model is used as the base model in this study.

However, the Bass model has several limitations that make it less applicable to new energy passenger vehicle diffusion. Firstly, the Bass model assumes that innovation remains static in the diffusion process, neglecting the impact of continuous technological advancements on product functionality improvement and cost reduction. As new energy vehicles evolve, the product value continues to improve, influencing both the diffusion rate and volume. Secondly, the Bass model treats diffusion solely as a function of time, ignoring intermediary factors such as product value, which influences consumer purchasing decisions. As the value of new energy vehicles increases over time, their relative advantages become more evident, stimulating consumer demand and accelerating diffusion. It can be assumed that new energy passenger vehicle diffusion is a time-dependent process mediated by product value, propagating among members of the social system. Finally, the Bass model assumes a constant market potential. In reality, the number of potential purchasers of new energy passenger vehicles grows as product value increases. Higher product value enhances consumer purchase intentions, thereby expanding the number of potential purchasers. Therefore, the Bass model needs to be improved to more accurately capture the diffusion dynamics of new energy passenger vehicles.

Existing studies have applied the Bass model to new energy passenger vehicle sales forecasting, often focusing on single factors such as charging infrastructure or price [10–14]. For example, Kumar et al. [12] considered the effect of the number of charging stations on electric vehicles in the Generalized Bass model, while Bitencourt et al. [10] considered the effect of price in the Bass model. However, these approaches fail to capture the multifaceted nature of consumer decision-making, which is influenced by multiple product attributes. Product value, reflecting consumers’ comprehensive evaluation of these attributes, serves as the foundation for purchase decisions [15]. Consumers purchase new energy passenger vehicles only when the product value aligns with their needs [16–17]. Despite its importance, the role of product value in shaping market potential and diffusion rates remains underexplored in the literature.

In this study, we propose and verify an Improved Bass Model based on Product Value (IBMPV) for forecasting new energy passenger vehicle sales. We first propose a product value function of new energy passenger vehicles based on the theory of value engineering. Second, considering that the number of potential adopters and their adoption rate increase with the enhancement of product value, we express market potential and diffusion rate as functions of the product value, respectively. Moreover, we further incorporate seasonal fluctuations to enable IBMPV to better capture real market dynamics. Finally, we use quarterly data on new energy passenger vehicles in China from 2014 to 2023 to verify the IBMPV and compare its predictive performance with the Bass, Gompertz, Logistic, and ARMAX model.

Our research has three main contributions. First, we improve the Bass model based on product value, which is an extension of the Bass model and provides a practical framework to better understand and predict new energy passenger vehicle diffusion. Second, we quantitatively estimate how product value impacts new energy passenger vehicle diffusion, which better reveals the mechanism of new energy passenger vehicle diffusion. Third, we demonstrate that technological innovation accelerates new energy passenger vehicle diffusion by enhancing both market potential and diffusion rates through product value, which is helpful for understanding the diffusion curve of new energy passenger vehicles and conducting further research on their sales forecasting.

The remainder of this paper is organized as follows. Section 2 reviews the research related to this study. Section 3 describes the IBMPV in detail. In Section 4, the predictive performance of IBMPV is evaluated and some comparisons with the Bass, Gompertz, Logistic, and ARMAX models are given. Finally, Section 5 discusses the conclusions, limitations, and potential directions for future research.

## 2. Literature review

### 2.1. Methods for forecasting new energy vehicle sales

Sales forecasting for new energy passenger vehicles is affected by multiple factors, with limited historical data available. Current studies typically categorize forecasting methods into two main types: micro-level and macro-level approaches. Micro-level methods commonly include the discrete choice model and agent-based model, while macro-level approaches primarily encompass machine learning techniques, the grey prediction method, the diffusion rate and time series method which includes Gompertz model, Logistic model, Bass model and its variations. Table 1 presents a comparison between this study and existing literature on new energy passenger vehicle sales forecasting, focusing on the methods used and the factors considered.

**Table 1:** Comparison between this work and previous literature on new energy vehicle sales forecasting.

Related work	Method	Product attributes considered					Product value
		Endurance	Charging infrastructure	Charging time	Power	Price	
Hackbarth and Madlener [18]	Discrete choice model	√	√	√		√	
Byun et al. [19]	Discrete choice model		√	√		√	
Klein et al. [5]	Agent-based model	√	√	√		√	
Wolf et al. [20]	Agent-based model	√		√		√	
Liu et al. [21]	Machine learning		√			√	
Ding et al. [22]	Machine learning	√			√	√	
Ding et al. [23]	Grey model						
Zeng et al. [2]	Grey model						
Li et al. [24]	Gompertz model						
Dhakal & Min [25]	Logistic model						
Bitencourt et al. [10]	Bass model					√	
Benvenutti et al. [26]	Generalize Bass model						
Kumar et al. [12]	Generalized Bass model		√				
Park et al. [27]	Generalized Bass model		√			√	
Xian et al. [28]	Generalized Bass model		√			√	
This work	Bass model	√	√	√	√	√	√

The discrete choice model focuses on the decision-making process of individual consumers, and subsequently identifies key factors influencing market penetration. For example, Hackbarth and Madlener [18] utilized discrete choice data to develop a mixed logit model, analyzing both the market demand potential and consumers’ willingness to pay for new energy vehicles in Germany. Similarly, Byun *et al*. 单击或点击此处输入文字。[19] used a discrete choice experiment to examine consumer vehicle preferences and forecast the dynamic market share of environmentally friendly vehicles. The agent-based model simulates complex systems by considering heterogeneous factors such as agent preference and behavior choice. For example, Klein *et al*. [5] developed an agent-based simulation to examine the purchasing behavior of German consumers concerning electric and plug-in hybrid electric vehicles. Similarly, Wolf *et al*. [20] employed an agent-based model to investigate the impact of policy interventions and social influence on consumers’ preferences for different modes of transportation. However, both the discrete choice model and agent-based model are used to investigate diffusion probability of new energy vehicles from the micro level of consumer choice, and most of the research is based on consumer-oriented questionnaire survey data, posing challenges for accurately forecasting new energy passenger vehicle sales.

Machine learning methods can effectively address complex issues influenced by multiple factors, making them suitable for sales forecasting of new energy vehicles. For example, Liu et al. [21] developed a multi-factor forecasting model that combines discrete wavelet transform and bidirectional LSTM to accurately predict new energy passenger vehicle sales. Ding et al. [22] introduced a hybrid prediction model that integrates recurrent neural network and long short-term memory neural network to forecast the sales of the BYD Tang. However, machine learning methods require large datasets for accuracy and often suffer from complexity and poor interpretability. In contrast, the grey prediction method performs well with small samples, and some studies have applied it to forecast new energy vehicle sales. For example, Ding et al. [23] developed a composite forecasting model that combines adaptive data pre-processing with an optimized nonlinear grey Bernoulli model, resulting in more accurate and robust predictions for new energy vehicle sales. Zeng et al. [2] proposed a novel grey prediction model with a variable structure, tailored to address the challenges posed by the small sample size of China’s new energy vehicle sales. However, grey prediction method cannot effectively incorporate the impacts of multiple factors on new energy vehicle diffusion, making it difficult to explain the diffusion mechanism.

In comparison, the diffusion rate and time series method uses diffusion rate and time series to understand the diffusion process, enabling better capture of the penetration patterns and adoption dynamics of new energy vehicles in the market, with strong interpretability and practicality. Li et al. [24] applied the Gompertz model and provincial data to analyze the non-linear relationship between private vehicle ownership and per capita GDP, revealing that the number of private vehicles owned in China ‘s provinces follows S-shaped development. Kumar et al. [12] used the Gompertz model, Logistic model, Bass model, and Generalized Bass model to forecast electric vehicle adoption in 20 countries, and the results showed that the fitting performance of these models for electric vehicle demand varied significantly across different countries. Park et al. [13] conducted a comparative analysis of Bass model, Logistic model, Gompertz model, and analogy method to forecast the market demand for hydrogen fuel cell vehicles. By varying the diffusion rates, they predicted the demand for hydrogen fuel cell vehicles under three scenarios by 2040, as well as the annual hydrogen demand and daily hydrogen demand per charging station, and the results showed that the Gompertz model is more suitable for predicting hydrogen fuel cell vehicle adoption. Dhakal & Min [25] analyzed the global diffusion of electric vehicles using the Logistic model and the Bass model, and they found that the Bass model fits the actual situation better than the Logistic model. Although these models are widely used in the study of new energy vehicle diffusion, the Gompertz model and the Logistic model are unable to analyze the impact of external variables such as infrastructure development, advancements in new energy vehicle-related technologies, and cost reductions. In contrast to the Gompertz model and the Logistic model, the parameters of the Bass model have clear economic significance, which can incorporate the influence of multiple factors and aids in explaining the model results and revealing the diffusion mechanisms of new energy vehicles. Therefore, the Bass model and its improvements are widely used for forecasting new energy vehicle sales.

The Bass model and its improvements for new energy vehicle sales forecasting often focus on considering the influence of specific factors, mainly focusing on infrastructure, purchase price and policy. For example, Bitencourt et al. [10] assumed that economic factors can significantly change the adoption of technologies such as electric vehicle, and improved Bass model based on Beck’s proposal that market potential is a function of simple payback time rather than a constant to assess the impact of public policy on electric vehicle diffusion in Brazil. Benvenutti et al. [26] considered a growing market as price decreases over time based on the Cobb-Douglas function and extended price as a decision variable to the Generalized Bass model, developing a system dynamics model to study the impact of public policy on the long-term diffusion of alternative fuel vehicles in Brazil. Kumar et al. [12] incorporated the impact of the number of charging stations as an external variable into the Generalized Bass model, and used the proposed model to forecast electric vehicle sales in 20 major countries. In addition, some studies extended the Generalized Bass model by simultaneously considering the impacts of cost reduction and infrastructure construction. For example, Park et al. [27] improved the Generalized Bass model by considering the impact of the number of hydrogen fueling stations and vehicle prices on hydrogen fuel cell vehicle sales as external variables, and proposed a penetration forecasting model. Xian et al. [28] developed a new energy vehicle ownership prediction method for China based on the Generalized Bass model, with the ratio of levelized cost of driving between fuel cell vehicles and internal combustion engine vehicles and the number of hydrogen refueling stations as external variables, in which levelized cost of driving includes the purchase cost and fuel cost of fuel cell vehicles, and the number of hydrogen refueling stations represents the level of infrastructure.

Previous studies have shown that the Bass model performs better in new energy vehicle sales forecasting due to its strong interpretability and lower data requirements. However, they mainly focused on specific factors, such as charging infrastructure and price. In reality, product attributes like driving range, charging infrastructure, price and cost have been identified as both drivers and barriers to new energy vehicle adoption, significantly influencing new energy vehicle sales [29,30]. Therefore, there is a notable gap in research that comprehensively considers the collective impact of these factors on new energy vehicle adoption. In this study, we develop a product value function and integrating it into the Bass model.

### 2.2. Studies on improving the Bass model

The Bass model suggests that potential consumers are influenced by external and internal factors when adopting innovation. This model is characterized by three parameters: m , p and q, where m represents the market potential, p denotes the external influence coefficient, and q is the internal influence coefficient [31]. However, the Bass model assumes that both market potential and influence coefficients remain constant, making it necessary to be improved in application. Table 2 illustrates that previous research has expanded upon these assumptions to enhance the Bass model. For example, Centrone et al. [32] introduced an exponential growth of market potential over time, accounting for the combined effects of economic and demographic factors. Dattée [33] improved the market potential in the Bass model by incorporating utility functions related to performance and price. Batista da Silva et al. [34] modeled the potential market with an approximate exponential shape over time in medium to long term projection by considering demographic and economic characteristics. Some studies improved the external and internal influence coefficients. For instance, Fan et al. [35] utilized the Naive Bayes algorithm to extract sentiment indices from online reviews and incorporated these into the internal influence coefficient of the Bass model, enhancing prediction accuracy for product sales forecasting. Similarly, Kim et al. [11] improved the external and internal influence coefficients by considering consumer web search behavior, actual data from search engine queries and new vehicle sales for each vehicle class and region are used to estimate the proposed model, and the prediction performance is compared with the Bass model and ARMAX model, validating the usefulness of data for internet search engine queries in forecasting new product diffusion. Moreover, some studies have improved both the market potential and the influence coefficients. For example, Zhang et al. [14] treated market potential as a linear function of the product macroeconomic index and modeled the external influence coefficient as a linear function of online search traffic. They used the S-curve function and online review data to estimate the internal influence coefficient. In another instance, Zhang et al. [36] developed a prediction method for movie box office performance, positing that the total number of potential buyers was related to the maximum number of microblog reviews. They modeled the external influence coefficient based on sentiment values, consumer RFM values, and attention to microblog reviews, while the internal influence coefficient was also a function of sentiment values.

**Table 2:** Comparison of this study with previous research on improved Bass models.

Related work	Factors considered	Improved market potential	Improved influence coefficients		Time-dependent	Product value-dependent
			External influence	Internal influence		
Centrone et al. [32]	Demographic and economic.	√			√	
Dattée [33]	Performance and price.	√			√	
Batista da Silva et al. [34]	Demographic and economic characteristics.	√			√	
Fan et al. [35]	Online review sentiment index.			√	√	
Kim et al. [11]	Consumer internet search behavior.		√	√	√	
Zhang et al. [14]	Product macroeconomic index; online search traffic; word-of -mouth index.	√	√	√	√	
Zhang et al. [36]	Sentiment value of microblog text; consumers’ RFM value; the degree of moviegoers’ attention.	√	√	√	√	
Kapur et al. [41]	Value and change point.					√
This work	The increase of product value	√				√

However, from a mathematical perspective, both the basic and the enhanced versions of the Bass model focus on the time-dependent aspects of the diffusion process. Specifically, they analyze how innovations propagate through the potential market over time via communication channels. In fact, apart from time, various decision variables, such as purchase cost and performance, influence the diffusion of a new product [37]. For example, Tsai [38] demonstrated that the diffusion of LCD TVs was influenced by price reductions, establishing that diffusion is price-dependent. While the Generalized Bass model expands the original Bass model by incorporating marketing factors such as price and advertising [39], it also maintains that diffusion is time-dependent, introducing time-based functions that define the rate of change in marketing efforts. These time-dependent functions serve to adjust the diffusion structure over time, accounting for delays in adoption that are influenced by time [40]. To address this limitation, some studies attempted to propose diffusion models that incorporate the key driving forces of diffusion. For example, Kapur et al. [41] proposed a value-based diffusion model by integrating the Bass model with a two-dimensional distribution function, which uses the Cobb-Douglas production function to combine the impact of the duration of the technology in the market and the changing price.

In summary, previous studies primarily improved the Bass model by relaxing the assumptions that diffusion parameters remain constant, mostly within a time-based framework. In contrast, our study believes that new energy vehicle diffusion is driven by product value and develops the diffusion model based on a product value framework. Product value, as the basis of purchasing decision for new energy vehicle consumers, significantly influences both market potential and diffusion rate. Therefore, constructing market potential and diffusion rate based on product value is a central focus of this study.

## 3. IBMPV building

### 3.1. Idea of IBMPV

#### 3.1.1. Proposal of product value function of new energy passenger vehicles.

As a substitute for traditional passenger vehicles, functionality and cost of new energy passenger vehicles are primary factors for consumers to make purchase decisions [17]. Consumers are often provided with information related to functionality and cost of new energy passenger vehicles to evaluate product value and make their purchasing decisions [42]. Moreover, improving functionality while controlling cost is more likely to boost consumer willingness to purchase than focusing on either aspect alone. Therefore, consumers’ purchase decisions at time t mainly depend on the relative value of functionality and cost of new energy passenger vehicles at that time. According to the theory of Value Engineering [43], the product value of new energy passenger vehicles can be defined as the ratio of the functionality provided to consumers when purchasing these vehicles to the cost incurred to obtain these functionalities. Eq. (1) provides the product value of new energy passenger vehicles at time t.


$$V_{t}=\frac{F_{t}}{C_{t}}$$ (1)

Where t represents the time since the introduction of new energy passenger vehicles into the market, measured in quarters. Given that the time boundary for this study spans from 2014 to 2023, we assume that new energy passenger vehicles are initially introduced into market in the first quarter of 2014, and each subsequent “t ” represents the progression of time in quarters, i.e., t=0,1,2,……,40. For example, t=0 corresponds to the initial introduction of new energy passenger vehicles in the first quarter of 2014; t=40 corresponds to the fourth quarter of 2023, representing the 40th quarter since the initial introduction of new energy passenger vehicles. $V_{t}$ indicates the product value of new energy passenger vehicles at time t, and $V_{t}>0$. $F_{t}$ indicates the function coefficient of new energy passenger vehicles at time t. $C_{t}$ denotes the cost coefficient of new energy passenger vehicles at time t.

(1) Function coefficient of new energy passenger vehicles

The functions of new energy passenger vehicles refer to the utility consumers derive from using the vehicles, primarily stemming from their physical characteristics or functional attributes, such as endurance performance, charging convenience, and power performance [44]. Endurance performance is an important indicator of technological advancement in new energy passenger vehicles, directly impacting consumers’ daily travel range. Limited endurance range has consistently been a significant barrier to new energy vehicle adoption and a primary cause of range anxiety [45]. Charging convenience plays a crucial role in extending the range of new energy passenger vehicles and reducing consumers’ range anxiety, directly influencing consumers’ use utility after purchasing new energy passenger vehicles. This is determined by both the number of charging infrastructure and the time for charging. On the one hand, augmenting the charging infrastructure significantly addresses consumers’ charging requirements, enhancing the overall convenience of new energy vehicle utilization. On the other hand, substantial reductions in charging duration due to technological advancements not only minimizes waiting time for vehicle owners, but also improves the efficiency of charging infrastructure, which also boosts the convenience of charging, thereby promoting new energy vehicle adoption. As an alternative to traditional passenger vehicles, new energy passenger vehicles are essentially tools for driving and traveling. Therefore, in addition to innovative attributes, power performance is a key factor that consumers are concerned about, as it is not only one of the most representative attributes of vehicles, but also a major weakness of new energy passenger vehicles [46]. Therefore, this study defines the functional attributes of new energy passenger vehicles from the consumer perspective by evaluating endurance, charging convenience, and power performance.

Given the varying importance of each functional attribute in consumer decision-making, it is necessary to assign weights to these functional attributes. In this study, the entropy weight method is employed to calculate the weights for each functional attribute due to its objectivity in weight assignment. Eq. (2) provides the functional relationship of new energy passenger vehicles at t time.


$$F_{t}=w_{R}\times R_{t}+w_{IC}\times IC_{t}+w_{EB}\times EB_{t}$$ (2)

Where $R_{t}$ denotes endurance performance of new energy passenger vehicles at time t; $IC_{t}$ denotes charging convenience of new energy passenger vehicles at timet; $EB_{t}$ denotes power performance of new energy passenger vehicles at time t. $w_{R}$ indicates the weight of endurance performance; $w_{IC}$ indicates the weight of charging convenience; $w_{EB}$ indicates the weight of power performance.

(2) Cost coefficient of new energy passenger vehicles

In the early stage of the new energy vehicle industry, many consumers take a wait-and-see attitude due to high purchase costs. In response to this phenomenon, subsidy policies are implemented by government to reduce the purchase cost, thus stimulating demands for new energy passenger vehicles. Despite the intensity of subsidies gradually decreases, technological advancements continue to lower innovation costs, resulting in a steady reduction in purchase prices [42]. However, the purchase cost remains a major concern for consumers. Fluctuations in cost not only directly affect consumers’ attitudes toward adopting new energy passenger vehicles but also influence overall diffusion [47]. In addition, as a kind of consumable goods, the operating cost is one of key factors for consumers to consider [48]. New energy passenger vehicles, powered by stable and low-cost electric energy, have a lower operating cost compared to traditional vehicles, which enhances their perceived value and increases purchase willingness. Therefore, we define the cost of new energy passenger vehicles as the sum of the purchase cost and the operating costs over the vehicle’s lifecycle. Here,$C_{t}$ represents the cost of new energy passenger vehicles at time t, as shown in Eq. (3).


$$C_{t}=P_{t}+U_{t}$$ (3)

Where $P_{t}$ represents the purchase cost of the new energy passenger vehicles at time t, and $U_{t}$ represents the operating cost of the new energy passenger vehicles at time t.

#### 3.1.2. Formation of dynamic market potential.

The market potential for new energy passenger vehicles increases with the improvement of technology and the reduction of prices [33]. During the diffusion process of new energy passenger vehicles, the continuous improvement of functionalities and the gradual reduction in purchase price, driven by technological innovation and economies of scale, contribute to ongoing enhancement of product value, which in turn constantly meets consumer demands for new energy passenger vehicles and increases their willingness to buy [42]. Therefore, it is essential to consider growth of market potential caused by the enhancement of product value, and improve the Bass model by modifying the assumption that market potential is constant. With reference to Centrone et al. [32], we assume that there is an exponential relationship between the market potential of new energy passenger vehicles and product value. Eq. (4) provides the market potential $m_{V_{t}}$ at time t.


$$m_{V_{t}}=m_{0}\times \exp(\alpha V_{t})$$ (4)

Where $m_{0}$ indicates the initial market potential of new energy vehicle market, α(α>0) is the influence coefficient of product value.

#### 3.1.3. Formation of the purchased group.

The Bass model believes that potential adopters take purchasing behaviors under the combined action of internal and external factors, ultimately resulting in the distribution of purchasing groups in the time dimension, as shown in Eq. (5).


$$n(t)=m\times f(t)=m\times \frac{p_{1}{\left(p_{1}+q_{1}\right)}^{2}e^{-\left(p_{1}+q_{1}\right)t}}{{\left(p_{1}+q_{1}e^{-\left(p_{1}+q_{1}\right)t}\right)}^{2}}$$ (5)

Where n(t) denotes the number of adopters at time t; m denotes the number of potential purchasers, i.e., the market potential; $p_{1}$ indicates the external influence coefficient; $q_{1}$ indicates the internal influence coefficient; f(t) denotes the proportion of potential purchasers converted to adopters at time t, i.e., the diffusion rate.

The Bass model represents innovation diffusion as a time-dependent process, without incorporating the mediating variables that influence diffusion. In fact, the ongoing enhancement of product value consistently meet consumer demands for new energy passenger vehicles, stimulating their purchase behavior and serving as the driving force of new energy passenger vehicle diffusion. That is, new energy passenger vehicle diffusion depends on the time-varying product value which is related to diffusion rate. Consequently, we assume that the diffusion rate of new energy passenger vehicles at time t is jointly determined by internal and external influences as well as the product value at time t. According to the Bass model, external influence stems from publicity, and internal influence comes from word-of-mouth, both of which remain constant during diffusion [49]. Therefore, it can be considered that the probability of potential buyers purchasing new energy passenger vehicles at time t is a function of product value, as shown in Eq. (6).


$$h\left(V_{t}\right)=\frac{f\left(V_{t}\right)}{1-F\left(V_{t}\right)}=p_{0}+q_{0}F\left(V_{t}\right)$$ (6)

Where $h(V_{t})$ represents the probability that a potential buyer who has not yet made a purchase at time t purchases due to the product value $V_{t}$ at time t. $f(V_{t})$ represents the proportion of purchasers to potential purchasers due to the product value $V_{t}$ at time t, that is, the diffusion rate. $F(V_{t})$ represents the proportion of cumulative purchasers to potential purchasers due to the product value $V_{t}$ at time t. $p_{0}$ and $q_{0}$ represents the external and internal influence coefficients, respectively.

By using F(0)=0, we can obtain the analytical solution of differential Eq. (5), which is expressed in Eq. (7).


$$F\left(V_{t}\right)=\frac{1-e^{-\left(p_{0}+q_{0}\right)V_{t}}}{1+\frac{q_{0}}{p_{0}}e^{-\left(p_{0}+q_{0}\right)V_{t}}}$$ (7)

The diffusion rate of new energy passenger vehicles at time t can be obtained by derivation of Eq. (6), as shown in Eq. (8).


$$f\left(V_{t}\right)=\frac{p_{0}{\left(p_{0}+q_{0}\right)}^{2}e^{-\left(p_{0}+q_{0}\right)V_{t}}}{{\left(p_{0}+q_{0}e^{-\left(p_{0}+q_{0}\right)V_{t}}\right)}^{2}}$$ (8)

### 3.2. Proposal of IBMPV

Informing consumers about product value of new energy passenger vehicles can enhance their understanding and awareness, thereby increasing their willingness to adopt new energy passenger vehicles. As product value accumulates and diffusion progresses, consumer purchasing behavior is stimulated, accelerating the diffusion rate of new energy passenger vehicles. Hence, we consider that the product value significantly impacts consumers’ purchase intentions and behaviors, which is reflected in the model as an impact on market potential and diffusion rate.

Moreover, new energy passenger vehicle sales in the fourth quarter are significantly higher than the first three quarters due to marketing activities, exhibiting obvious seasonal fluctuations [2,21]. To more accurately forecast new energy passenger vehicle sales, we model the seasonal fluctuations as the influence of marketing activities on the conversion of market potential into purchased group in the fourth quarter [3,50], and denoted by a seasonal coefficient $sea_{t}$, as shown in Eq. (9). Here, the fourth quarter can be expressed as $Q_{4}$, while other quarters can be expressed as$Q_{0}$;$mean_{sale_{Q_{4}}}$ denotes the average sales of all the fourth quarters, $mean_{sale_{Q_{0}}}$ indicates the average sales of all quarters except all the fourth quarters.


$$\;sea_{t}=\left\{ \begin{array}{cc} & \frac{\;mean_{sale_{Q_{4}}}}{\;mean_{sale_{Q_{0}}}},t=4n-1(n\in N^{+})\\ & 1,t\neq 4n-1(n\in N^{+}) \end{array}\right.$$ (9)

Therefore, in this study, new energy passenger vehicle diffusion is considered as a function of product value. The dynamic market potential of new energy passenger vehicles at time t is transformed into the adoption of new energy passenger vehicles at time t under the combined action of internal influence, external influence and seasonal coefficient, as shown in Eq. (10).


$$\begin{array}{c} n\left(V_{t}\right)=m_{V_{t}}\times f\left(V_{t}\right)\times {\text{sea}}_{t} \end{array}$$ (10)

Substituting Eqs. (4) (8) (9) into Eq. (10), the IBMPV can be obtained as follows.


$$n\left(V_{t}\right)=m_{0}\times e^{\alpha V_{t}}\times \frac{p_{0}{\left(p_{0}+q_{0}\right)}^{2}e^{-\left(p_{0}+q_{0}\right)V_{t}}}{{\left(p_{0}+q_{0}e^{-\left(p_{0}+q_{0}\right)V_{t}}\right)}^{2}}\times {\mathrm{sea}}_{t}$$ (11)

### 3.3. Characteristics of IBMPV

**Fig 1:**
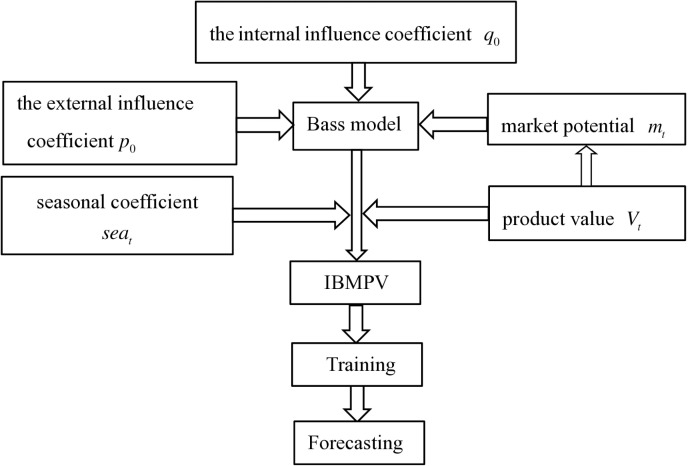
The relationship between IBMPV and the Bass model.

The IBMPV proposed in this paper is an improvement based on the Bass model, and the relationships between its variables are shown in Fig 1. The characteristics of IBMPV are analyzed through a comparison with the Bass model as follows.

(1) Compared with the static market potential in the Bass model, IBMPV considers the increase in the number of potential adopters driven by the enhanced product value of new energy passenger vehicles, achieving dynamic market potential.
(2) The Bass model assumes that innovation spread through the social system over time which is related to the diffusion rate, and takes diffusion as a function of time. In contrast, IBMPV assumes that new energy passenger vehicle diffusion is a process that spreads over time among members of the social system with product value as an intermediary, and the new energy passenger vehicle diffusion is expressed as a function of product value which is related to the diffusion rate.
(3) Compared with the Bass model, IBMPV introduces the seasonal coefficient to describe the impact of seasonal fluctuations on new energy passenger vehicle sales, which better reflects the real market conditions and improves prediction accuracy, making it more suitable for forecasting with quarterly data.
(4) By incorporating product value into the Bass model, IBMPV accurately captures the relationship between product value evolution and new energy passenger vehicle diffusion, which helps to explain the causes and trends of new energy passenger vehicle diffusion. For example, the enhancement in product value can promote a significant increase in the number of potential adopters, providing more insights for decision-makers.

## 4. Performance evaluation of IBMPV

### 4.1. Data collection

According to the China Association of Automobile Manufacturers, China’s new energy vehicle industry transitioned from the introduction phase to a growth phase in 2014, with private consumption increasingly becoming the primary driver in the new energy vehicle market. Consequently, this study utilizes quarterly data from 2014 to 2023 on sales, functionalities, and costs for parameter estimation and model validation. Specifically, sales data are sourced from the China Association of Automobile Manufacturers(http://www.caam.org.cn/); the number of charging stations is obtained from the China Electric Vehicle Charging Infrastructure Promotion Alliance(http://www.evcipa.org.cn); the price of electricity comes from the China Power(http://www.chinapower.com.cn/); and data on other functional attributes and purchase prices are collected from Autohome(https://www.autohome.com.cn/). The relevant variables and data sources for new energy passenger vehicles are listed in Table 3.

**Table 3:** Variables and data sources.

Variables	Sources	Explanation
the New European Driving Cycle (NEDC) range standard $R_{t}$	Autohome	
the number of public charging piles $NI_{t}$	China Electric Vehicle Charging Infrastructure Promotion Alliance	Convert the statistical data units into ten thousand
the maximum power $MP_{t}$	Autohome	
the battery capacity $CB_{t}$	the Autohome	
the power consumption of 100 kilometers $E_{t}$	Autohome	
purchase cost $P_{t}$	Autohome	Considering the role of the subsidy policy, take the manufacturer’s guidance price after considering the subsidy on the Autohome.
The price of electricity $P_{e}$	China Power Grid	Due to the relatively small fluctuations in electricity prices, the unit price of electricity is assumed to be 0.585 yuan/kW·h.
the lifecycle mileage ML	Autohome	According to Yang et al. [48], it is assumed that the life cycle mileage of new energy passenger vehicles is 150,000 kilometers
Sales of new energy passenger vehicles	China Association of Automobile Manufacturers	Monthly sales data from 2014 to 2023 are collected and consolidated into quarterly sales data in 10000 units.

For functional attributes and price data of new energy passenger vehicles, this study collects data on all types owned by ten major manufacturers from 2014 to 2023, including BYD, SGMW, Tesla (China), Aion, China Changan Automobile Group, Chery New Energy, Geely Auto, NIO, Ideal, and Xiaopeng Motors. The reason for selecting these top ten manufacturers to represent the new energy passenger vehicle market is that they are leading companies in the new energy vehicle industry, recognized by consumers as prominent new energy vehicle brands. These manufacturers include not only traditional automakers but also emerging players in the new energy vehicle sector. Moreover, their combined market share accounts for approximately 69.4% of the new energy passenger vehicle market, making them highly representative. A total of 1187 records for new energy passenger vehicles are collected, each including attributes such as time to market, energy type, vehicle level, manufacturer’s guidance price, NEDC endurance mileage, maximum power, battery capacity, the power consumption of 100 kilometers, and the lifecycle mileage. After excluding 133 records with incomplete information, the remaining 1054 records are used for parameter estimation and model fitting. The data includes battery electric vehicles and plug-in hybrid electric vehicles categorized by energy type, as well as four vehicle classes: sedan, SUV, MPV, and minibus. The data for all types launched in each quarter are obtained according to the launch time of new vehicles, and then the attribute values of new energy passenger vehicles in each quarter are obtained by weighted average according to the energy type and vehicle class. Finally, the function coefficient and price coefficient for 40 quarters are obtained. We designated data from 32 quarters as the training set and data from 8 quarters as the test set.

Referring to the research of Massiani and Gohs [51], we estimate the market potential for new energy passenger vehicles by considering their penetration rate in passenger vehicle market and the total passenger vehicle ownership. As we specify the first quarter of 2014 as the initial date, the initial market potential for the new energy vehicle market $m_{0}$ is calculated as 0.1% of the national vehicle ownership at the end of 2013, which amounts to approximately 48.7259 ten thousand. Moreover, according to Yang et al. [48], we assume that the lifecycle mileage of new energy passenger vehicles is 150,000 kilometers.

### 4.2. Selection of variables

(1) Endurance performance

In China, the New European Driving Cycle (NEDC) range standard is the official benchmark for evaluating the comprehensive mileage of new energy passenger vehicles. Therefore, we evaluate the endurance performance using NEDC range data from Autohome and apply normalization to address potential scale inconsistencies.

(2) Charging convenience

The charging convenience of new energy passenger vehicles depends on the number of charging infrastructures and the charging time. It can be quantified as the service capacity of charging infrastructures per unit time, defined as the ratio of the number of charging infrastructures to the energy supply time. In this study, we measure the level of charging convenience as the ratio of the number of public charging piles to the time required for fast charging. Assuming that fast charging for new energy passenger vehicles takes 30 minutes, the number of charging infrastructures is converted into the level of charging convenience using Eq. (12), and the level of charging convenience is then normalized.


$$IC_{t}=\frac{NI_{t}}{30}$$ (12)

Where $IC_{t}$ indicates the charging convenience at time t, $NI_{t}$ indicates the number of public charging piles at time t.

(3) Power performance

Power performance of new energy passenger vehicles can be quantified by the ratio of the battery’s maximum power to its capacity. The maximum power influences both the ability to accelerate in the mid-to-late-stage and the maximum speed, while battery capacity represents the energy storage capacity of the vehicle, and a larger capacity results in an extended driving range. The ratio can be defined as the battery efficacy ratio, representing the maximum power generated per kilowatt-hour of battery capacity. A higher efficiency ratio indicates better battery efficiency and superior vehicle power performance. The battery efficacy ratio at time t is given by Eq. (13), followed by a normalization process.


$$EB_{t}=\frac{MP_{t}}{CB_{t}}$$ (13)

Where $EB_{t}$ denotes the battery efficacy ratio at time t, $MP_{t}$ denotes the maximum power of battery at the time t, and $CB_{t}$ denotes the battery capacity at the time t.

(4) Cost

The cost of new energy passenger vehicles at time t is the sum of purchase cost and operating cost, as shown in Eq. (3), and then normalization is performed. For the purchase cost of new energy passenger vehicles at time t, considering the role of subsidy policies after 2014, we use the manufacturer’s suggested retail price adjusted for subsidies from Autohome, that is, the purchase price after the implementation of subsidies. And then normalization is performed. Moreover, following the work of Yang et al. [48], the operating cost of automobile is determined by the lifecycle mileage, energy efficiency and fuel price. The operating cost of new energy passenger vehicles $U_{t}$ is calculated using Eq. (14).


$$U_{t}=P_{e}\times E_{t}\times ML$$ (14)

Where $P_{e}$ is the unit price of electricity, $E_{t}$ is the power consumption efficiency of new energy passenger vehicles at time t, that is, the power consumption of 100 kilometers, and ML is the lifecycle mileage.

### 4.3. Calculation of variables

(1) Function coefficient

Before calculating function coefficients of each quarter, the entropy weight method is used to obtain weights of each functional attribute. The specific calculation procedure is as follows.

1) An index matrix is constructed, as shown in Eq. (15). Suppose that there are T quarters and J functional attributes, $x_{tj}$ represents the value of the j th functional index in the t th quarter.

$$X={\left(x_{tj}\right)}_{T\times J},\;(t=1,2,\ldots \ldots ,T;j=1,2,\ldots \ldots ,J)$$ (15)

2) Since the functional attributes of new energy passenger vehicles are all positive indicators, the extreme value method is used for dimensionless, as shown in Eq. (16).

$$X_{tj}^{\prime }=\frac{X_{tj}-m_{j}}{M_{j}-m_{j}}$$ (16)

In Eq. (16), $m_{j}$ and $M_{j}$ represent the minimum and maximum values of the corresponding index for the j th functional attribute, respectively.

3) In order to eliminate the influence of zero, the matrix is shifted to the right by 0.0001 units.

$$X_{t}^{j\prime \prime }=X_{tj}^{\prime }+0.0001$$ (17)

4) The j th functional attribute in the t th quarter are shown in Eq. (18).

$$P_{tj}=\frac{X_{t}^{j\prime \prime }}{\sum_{t=1}^{T}X_{t}^{j\prime \prime }},\;0\leq P_{tj}\leq 1$$ (18)

5) Calculate the entropy of the j th functional attribute, as shown in Eq. (19).

$$e_{j}=-\frac{1}{\ln T}\sum_{t=1}^{T}P_{tj}\ln P_{tj}$$ (19)

6) Calculate the difference coefficient of the j th functional attribute, as shown in Eq. (20).

$$g_{j}=1-e_{j},\;0\leq e_{j}\leq 1$$ (20)

7) Calculate the weight of the j th functional attribute, as shown in Eq. (21). The greater the index coefficient obtained by the entropy weight method, the greater the weight, the greater the contribution to the results.

$$w_{j}=\frac{g_{j}}{\sum_{j=1}^{J}g_{j}}$$ (21)

The results of weight calculation are as follows: 0.2409 for endurance performance, 0.5553 for charging convenience, and 0.2038 for power performance. Using the functional attribute values and weights of 36 quarters, the function coefficient of each quarter of new energy passenger vehicle market can be calculated according to Eq. (2).

(2) Product value

**Fig 2:**
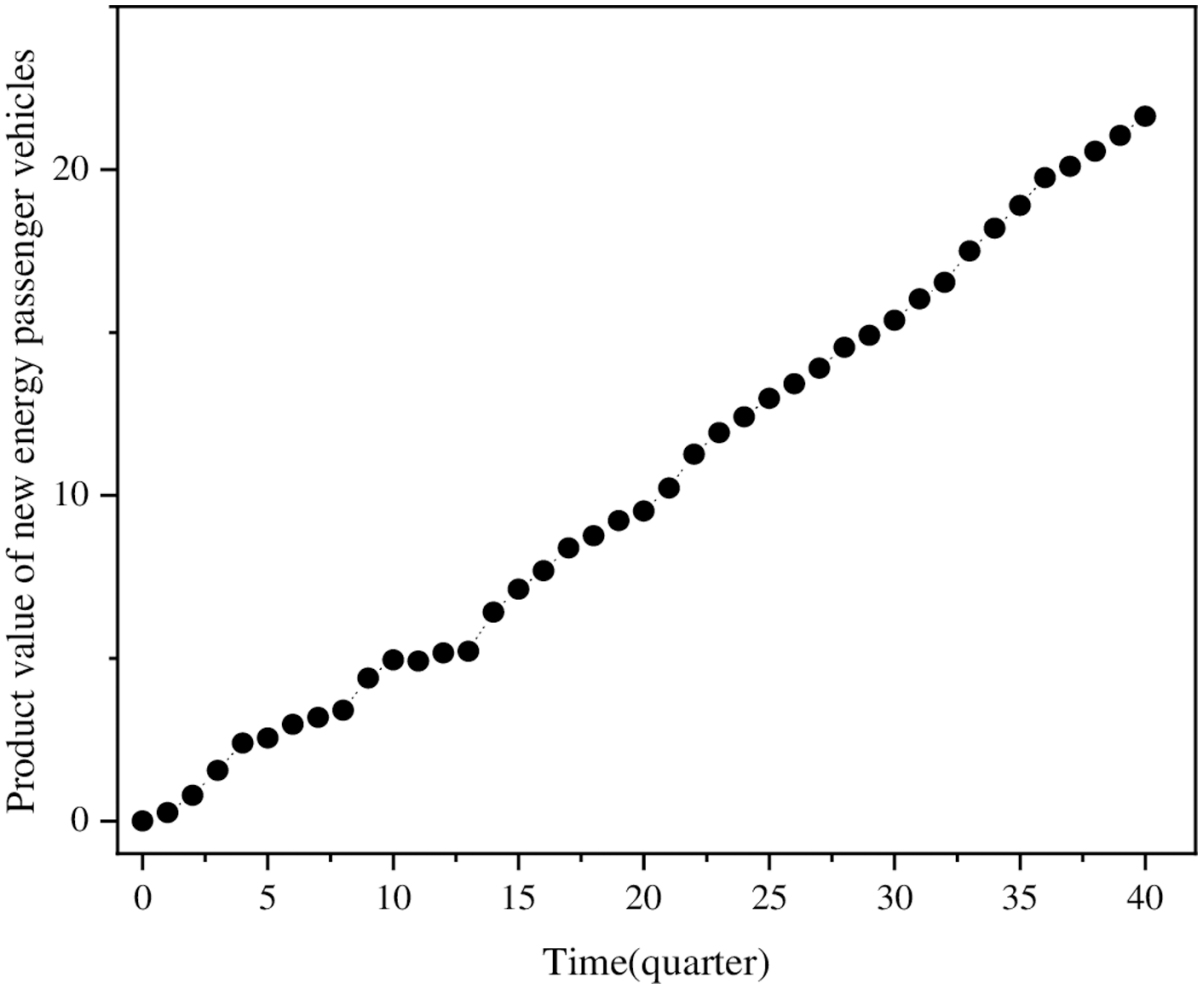
The trend of product value of new energy passenger vehicles over time.

Using the function and cost coefficients for each quarter derived from 1054 data points on new energy passenger vehicles, the product value for each quarter is calculated according to Eq. (1). Fig 2 depicts the trend of product value of new energy passenger vehicles over time. Overall, product value of new energy passenger vehicles exhibits an increasing trend, which is in line with the general principle that product value increases with technological progress during innovation diffusion [15,43]. In the early stages of industrial development, the limited variety of vehicle types significantly influences on product value, especially when specific types dominate the market, resulting in fluctuations in product value growth. As the market diversifies and technological advancements stabilize, product value grows steadily.

(3) Seasonal coefficient

Using the quarterly sales data of new energy passenger vehicles from the training set, the seasonal coefficient of the new energy vehicle market is calculated to be 2.18 by Eq. (9).

### 4.4. Predictive performance evaluation of IBMPV

Both Gompertz model and Logistic model are typical S-curve growth models, which are widely used in the study of new energy vehicle diffusion [12–13]. However, these traditional S-curve models fall short in capturing external factors and dynamic characteristics. In contrast, the ARMAX model, by integrating the properties of Autoregressive (AR), Moving Average (MA), and exogenous variables (X), is capable of effectively capturing the dynamic features of time series and explicitly incorporating the impact of external factors on new energy passenger vehicle diffusion. Therefore, we compare the Bass, Gompertz, Logistic and ARMAX models with the proposed IBMPV. We first estimate the model parameters using the training dataset, then predict the market potential and sales volume in the test set, and finally evaluate the performance of IBMPV by comparing the five models.

#### 4.4.1. Analysis of parameters.

We use the new energy passenger vehicle data of 32 quarters in the training set to fit the model parameters in Eq. (11), including the influence coefficient of product value α, the external influence coefficient $p_{0}$, the internal influence coefficient$q_{0}$. The objective function is constructed as $\min\sum_{t=0}^{T}{\left(n(V_{t})-\hat{n}(V_{t})\right)}^{2}$, where T representsthe diffusion time of new energy passenger vehicles, $n(V_{t})$ represents the actual sales of new energy passenger vehiclesat time t, $\hat{n}(V_{t})$ represents the predicted sales of new energy passenger vehicles at time t according to the model proposed in Eq. (11). The quasi-Newton algorithm as presented in R4.1.3 is used to optimize the objective function, and the asymptotic F distribution of nonlinear regression is used to test the significance of the parameters. The results of parameters estimation are presented in Table 4.

**Table 4:** Results of parameter estimation and significance testing.

Parameters	α	$p_{0}$	$q_{0}$
Estimated value	0.23	0.007	0.148
p-value	0.00^***^	0.00^***^	0.00^***^

Note:

*** denotes significance at p = 0.00,

** denotes significance at p = 0.01, and

* denotes significance at p = 0.05.

The goodness of fit based on the training set shows $R^{2}=0.851$, which indicates that the model fits very well. As shown in Table 4, the product value influence coefficient α is significant at the level of p=0, highlighting that the product value of new energy passenger vehicles plays a key role in shaping market potential, consistent with the reality. The internal influence coefficient $q_{0}$ is significantly larger than the external influence coefficient $p_{0}$, aligning with general principles of innovation diffusion. This suggests that, in the development of the new energy vehicle industry, internal influences such as word-of-mouth have a greater impact on consumer purchase behavior than external influences like mass media. This finding aligns with Kumar et al. [12] and Park et al. [13], as well as the real-world dynamics of new energy passenger vehicle diffusion, This is because some factors, such as convenience and safety, which are not easily perceived directly by consumers and rely on word-of-mouth among consumers to spread, play a crucial role in the adoption of new energy passenger vehicles. In the early stage of new energy vehicle industry, the limited number of innovators leads to relatively slower diffusion. However, as product value increases and the number of innovators grows, the influence of product value and imitation effect become pronounced. This significantly boosts consumer awareness and acceptance of new energy passenger vehicles, driving rapid diffusion among potential adopters.

#### 4.4.2. Comparison of the market potential.

**Fig 3:**
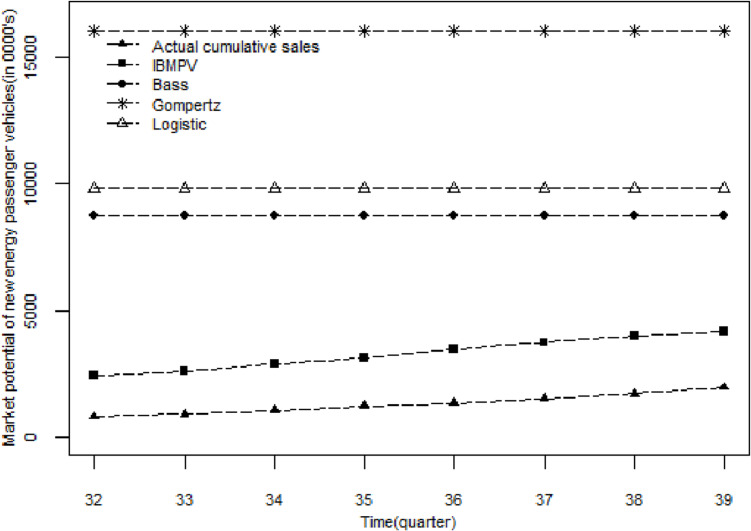
Comparison of market potential of new energy passenger vehicles in test set.

The ARMAX model focuses more on sales forecasting rather than market potential. Therefore, in this section, we only compare the prediction performance of the IBMPV on market potential with the Bass, Gompertz, and Logistic models. Fig 3 illustrates the trends over time for the dynamic market potential predicted by the IBMPV, the market potential in the Bass, Gompertz, and Logistic models, and the actual cumulative sales in the test set. The figure clearly demonstrates that, according to IBMPV in Eq. (11), the product value of new energy passenger vehicles experiences a gradual increase over time, leading to a corresponding growth in the market potential. In the early stages of industrial development, slow technological progress and limited maturity hinder consumer willingness to buy, resulting in slow market potential growth. However, with the acceleration of technological progress and the increasing adoption of new energy passenger vehicles, consumer demands are consistently met, awareness of new energy passenger vehicles continues to rise, and purchase intentions are significantly strengthened, thereby accelerating the expansion of market potential.

Table 5 compares the prediction accuracy of IBMPV, Bass, Gompertz, and Logistic models for new energy passenger vehicle market potential in the test set. According to Eq. (4) and the parameters in Table 4, we can obtain the new energy vehicle market potential for eight quarters in the test set. According to Table 5, the relative error between the market potential predicted by IBMPV and the actual cumulative sales is significantly lower than that of the other three models. Therefore, IBMPV is more applicable to new energy passenger vehicle diffusion than the three comparison models.

**Table 5:** Comparison of prediction accuracy of new energy passenger vehicle market potential in test set.

Time (quarter)	Predicted value (in 0000’s) & Relative error	IBMPV	Bass	Gompertz	Logistic	Actual cumulative sales
2022-Q1	Predicted value	2431.37	8753.75	16013.22	9816.66	809.09
	Relative error	200.51%	981.93%	1879.16%	1113.30%	
2022-Q2	Predicted value	2605.06	8753.75	16013.22	9816.66	917.89
	Relative error	183.81%	853.68%	1644.57%	969.48%	
2022-Q3	Predicted value	2889.13	8753.75	16013.22	9816.66	1063.89
	Relative error	171.56%	722.81%	14051.6%	822.71%	
2022-Q4	Predicted value	3131.32	8753.75	16013.22	9816.66	1233.39
	Relative error	153.88%	609.73%	1198.31%	695.91%	
2023-Q1	Predicted value	3464.80	8753.75	16013.22	9816.66	1356.49
	Relative error	155.42%	545.32%	1080.49%	623.68%	
2023-Q2	Predicted value	3738.02	8753.75	16013.22	9816.66	1527.09
	Relative error	144.78%	473.23%	948.61%	542.83%	
2023-Q3	Predicted value	3986.67	8753.75	16013.22	9816.66	1725.99
	Relative error	130.98%	407.17%	827.77%	468.76%	
2023-Q4	Predicted value	4155.18	8753.75	16013.22	9816.66	1963.89
	Relative error	111.58%	345.74%	715.38%	399.86%	

#### 4.4.3. Comparison of prediction results.

**Fig 4:**
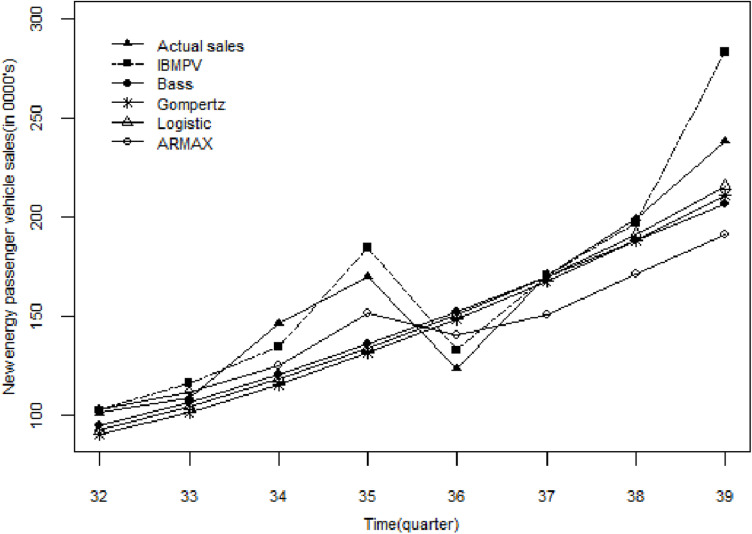
Comparison of verification results of new energy passenger vehicle sales in test set.

Table 6 compares the fitting performance of the IBMPV, Bass, Gompertz, Logistic and ARMAX models on the test set. As shown in Table 6, the goodness of fit $R^{2}$ for IBMPV surpasses that of the other four models. Additionally, both RMSE and MAPE for IBMPV are lower than those of the comparison models, indicating that IBMPV demonstrates the best predictive performance among these models. By substituting the parameters from Table 4 and the market potential from Table 5 into Equation (11), we obtain the sales volume of new energy passenger vehicles for the eight quarters in the test set. The predicted results of IBMPV, Bass, Gompertz, Logistic and ARMAX model for new energy passenger vehicle sales are shown in Fig 4, with the corresponding prediction accuracy presented in Table 7. As illustrated in Fig 4, the sales of new energy passenger vehicles have shown a rapid growth trend in recent years. Compared to the four comparison models, IBMPV fits the sales curve of new energy passenger vehicles more accurately, particularly in capturing seasonal fluctuations, which enables IBMPV to better forecast the diffusion trend of new energy passenger vehicles, thereby providing a valuable foundation for enterprises in formulating production and marketing strategies. As shown in Table 7, overall, IBMPV exhibits a smaller relative error in predicted results compared to the four comparison models, confirming that IBMPV is more suitable for forecasting new energy passenger vehicle sales.

**Table 6:** Comparsion in terms of fitting effect of the test set.

	$R^{2}$	RMSE	MAPE
IBMPV	0.837	17.696	0.061
Bass	0.754	21.751	0.120
Gompertz	0.735	22.562	0.141
Logistic	0.752	21.927	0.123
ARMAX	0.709	23.669	0.124

**Table 7:** Comparison of prediction accuracy of new energy passenger vehicle sales in test set.

Time (quarter)	Predicted value (in 0000’s) & Relative error	IBMPV	Bass	Gompertz	Logistic	ARMAX	Actual sales
2022-Q1	Predicted value	102.2	94.0	90.3	92.1	102.3	100.8
	Relative error	1.39%	-6.75%	-10.42%	-8.63%	1.49%	
2022-Q2	Predicted value	115.8	106.6	101.2	104.3	111.5	108.8
	Relative error	6.43%	-2.02%	-6.99%	-4.14%	2.48%	
2022-Q3	Predicted value	134.1	120.5	115.4	118.0	125.0	146.0
	Relative error	-8.15%	-17.47%	-20.96%	-19.18%	-14.38%	
2022-Q4	Predicted value	184.1	135.7	131.1	133.4	151.2	169.5
	Relative error	8.61%	-19.94%	-22.65%	-21.30%	-10.80%	
2023-Q1	Predicted value	132.5	152.0	148.3	150.5	139.8	123.1
	Relative error	7.64%	23.48%	20.47%	22.26%	13.57%	
2023-Q2	Predicted value	170.3	169.4	167.3	169.7	150.5	170.6
	Relative error	-0.18%	-0.70%	-1.93%	-0.53%	-11.78%	
2023-Q3	Predicted value	196.6	187.7	188.2	191.2	170.7	198.9
	Relative error	-1.16%	-5.63%	-5.38%	-3.87%	-14.18%	
2023-Q4	Predicted value	282.7	206.6	211.0	215.3	190.8	237.9
	Relative error	18.83%	-13.16%	-11.31%	-9.50%	-19.80%	

#### 4.4.4. Comparison of internal and external influence coefficients.

Table 8 presents the internal and external influence coefficients for both the IBMPV and Bass models. In the Bass model, the external influence coefficient $p_{1}$ of new energy passenger vehicle diffusion reaches its lower limit, while the internal coefficient $q_{1}$ significantly exceeds the external coefficient $p_{1}$. This suggests that, under assumptions of the Bass model, potential buyers are primarily influenced by internal factors, with external influences considered negligible, which contradicts reality. In contrast, the IBMPV demonstrates both the internal influence coefficient and the external influence coefficient within reasonable limits, with the external influence coefficient is smaller than the internal influence coefficient, i.e., $p_{0}\;<\;q_{0}$, indicating that in the early stages of new energy vehicle development, potential adopters are mainly influenced by external factors such as mass media; however, as new energy passenger vehicle diffusion progresses, the role of external influence on potential adopters diminishes, while the significance of internal influence, such as word-of-mouth, become more significant. This aligns with the reality and supports the validity of IBMPV. Moreover, the external influence coefficient of IBMPV is greater than that of the Bass model, while its internal influence coefficient is smaller, suggesting that under the influence of product value, external influences have a greater impact on potential adopters, while internal influences have a reduced effect on potential consumers’ adoption of new energy passenger vehicles.

**Table 8:** Comparison of internal influence coefficient and external influence coefficient.

	External influence coefficient	Internal influence coefficient
IBMPV	0.007	0.148
Bass	0.0001	0.151

### 4.5. Discussion

Our study demonstrates that the IBMPV model outperforms the Bass model in both fitting accuracy and predictive performance. The Bass model assumes that innovation remains constant throughout the diffusion process and that the market potential is fixed, thereby neglecting the impact of factors such as advancements in new energy passenger vehicle technology on diffusion and total market size. Additionally, the Bass model treats diffusion solely as a function of time, without considering intermediate factors such as product value. To address these limitations, we propose the IBMPV model, which incorporates product value as a key factor in new energy passenger vehicle diffusion, making it better suited for new energy passenger vehicle diffusion. Firstly, Kapur et al. [41] considered new product sales as a function of product value, developed a value-based diffusion model by integrating the Bass model with a two-dimensional value function, and verified that the value-based model has better forecasting efficiency than the basic Bass model. Similarly, we express the diffusion of new energy passenger vehicles as a function of product value, but we further consider the impacts of product value on market potential and propose a dynamic market potential that grows exponentially with product value. Unlike Kapur et al. [41], who employed the Cobb-Douglas production function to represent product value based on time and price fluctuations, we derive the product value function for new energy passenger vehicles from value engineering theory, focusing on functionality and cost, which are key factors influencing consumer decisions. Secondly, to enhance the accuracy of model parameter estimation, we build on Centrone et al. [32] by relaxing the assumption of constant market potential in the Bass model, and separately estimate the market potential and adoption of new energy passenger vehicles. While Centrone et al. [32] suggested that market potential grows exponentially over time, their model attributes this growth solely to time. In contrast, Adner and Levinthal [52] argued that consumer purchasing decisions fluctuate with evolving product performance and price, with market space defined by both functionality and price. Following this, we model market potential as a function of product value, reflecting its growth over time. This approach aligns with Dattée [33], who found that market potential increases with technological advancements and price reductions.

It is worth noting that the relative error of IBMPV is larger than that of the Bass, Gompertz, and Logistic models in the fourth quarter of 2023, which is may caused by three reasons. Firstly, with the maturity of new energy passenger vehicle technology, consumer awareness and acceptance have continued to improve, and consumers’ purchase decisions have become more rational, which reduces the seasonal fluctuation of the new energy passenger vehicle market. Secondly, as new energy passenger vehicles become increasing diverse, the market competition has gradually intensified, and automakers have balanced their sales throughout the year through promotional activities, reducing seasonal fluctuations. Finally, policies in new energy vehicle sector have shifted from direct subsidies to infrastructure construction and long-term planning, diminishing the short-term impact on market demand and leading to a more stable new energy vehicle market. In summary, it is the result of a combination of factors, including market maturity, changes in consumer behavior, and policy stabilization. It also points the way for our future research, that is, the need to further develop dynamic seasonal coefficients to better capture the seasonal fluctuations in the new energy vehicle market.

Based on the results, we present the following findings. First, IBMPV demonstrates better prediction accuracy for both market potential and adoption compared with the Bass model, confirming that new energy passenger vehicle diffusion depends on product value, which is consistent with the view that product sales behavior is a function of the value of products proposed by Kapur et al. [41]. Second, market potential grows exponentially with increasing product value, a result consistent with Wirges et al. [53], who demonstrated that the growth of new energy vehicle sales follows an exponential distribution. This finding suggests that new energy passenger vehicles with higher product value are more attractive to consumers, accelerating the expansion of market potential for new energy vehicles and fostering widespread adoption. For new energy passenger vehicles, high product value typically reflects enhanced functionality and lower costs. Therefore, new energy vehicle enterprises need to enhance product value through continuous innovation, thereby attracting more potential adopters and promoting new energy passenger vehicle diffusion. Furthermore, when analyzing internal and external coefficients, it is clear that internal factors, such as word-of-mouth, play a more significant role than external factors like mass media ($q_{0}>p_{0}$), consistent with the findings of Kumar et al. [12] and Park et al. [13]. Moreover, the external influence coefficient in the IBMPV model is higher than that in the Bass model. This is because the increase in product value enhances the appeal of new energy passenger vehicles, encouraging adoption among innovators.

However, we obtain a counterintuitive result in terms of the internal influence coefficients. That is, the internal influence coefficient of IBMPV is smaller than that of the Bass model, suggesting that internal influences, such as word-of-mouth, weaken under the influence of product value. This phenomenon can be explained by three main reasons. Firstly, in emerging markets, consumers prioritize product value, as they have not yet developed a stable perception of product reputation. As the market matures, consumers become more precise in product requirements and standards, and they can more directly obtain objective data from product specifications, lab tests, and independent evaluations, reducing their reliance on word-of-mouth. Secondly, Shi et al. [54] noted that the development of the new energy vehicle industry is significantly influenced by policy and innovation factors. With the reduction of policy support, consumers pay more attention to product attributes. Particularly, with advancing technology and evolving product attributes, the product value is continuously enhanced, making it more attractive to consumers, rather than relying on word-of-mouth. Thirdly, emerging products often suffer from limited information and high uncertainty, leading consumers to rely more on product value than word-of-mouth when making purchase decisions. These factors collectively contribute to a smaller internal influence coefficient, highlighting the growing importance of product value in consumer decision-making. Therefore, continuous technological innovation is essential to attract more early adopters and imitators. Particularly for imitators, enterprises and governments can encourage adopters to share their experiences by improving customer service and creating online communities to amplify word-of-mouth effects.

Although our work presents several findings, there are some limitations that need to be addressed. First, the product value in this study mainly focuses on the functional values of new energy passenger vehicles, which is regarded as key factors in consumer choice, including performance, convenience and monetary value. However, according to consumption value theory, consumers’ perception of value also encompasses non-functional aspects, such as emotional, social and epistemic values [17], which are not considered in this study. Future research can incorporate both functional and non-functional values. Second, the competition of traditional fuel vehicles, which affects the sales of new energy passenger vehicles, is not considered in this paper. Traditional fuel vehicles still dominate the automotive market in many regions, and their pricing, performance, and infrastructure advantages may significantly influence consumers decisions. Some studies suggested that there is competition and inhibition between traditional fuel vehicles and new energy vehicles, with growth in one potentially limiting the other’s market share单击或点击此处输入文字。 [54,55].The market evolution and dynamic competition between them are similar to the interspecies competition in ecology, and the Lotka-Volterra model is used to study the market evolution. While Xian et al. [28]used the Bass model to examine the impact of traditional fuel vehicles on new energy vehicle diffusion, most focus only on cost comparisons, neglecting the comparison of multiple product attributes from the perspective of product value. In fact, consumers will choose passenger vehicles with competitive advantages by comparing the product value. In the future, we will incorporate the competition of traditional fuel vehicles into the new energy passenger vehicle diffusion model from the perspective of product value. Finally, the data on new energy vehicle attributes used in this study are obtained from new energy vehicle websites, which may not fully capture consumer preferences and behaviors. In the future research, we can enhance the analysis by incorporating additional data sources, such as consumers Internet search data or online reviews, to better capture consumer sentiment, preferences, and emerging trends, improving the accuracy and relevance of the model.

## 5. Conclusion

In this study, we propose the product value function of new energy passenger vehicles based on the theory of value engineering, by considering the functions and costs that consumers pay primary attention to. On this basis, we improve the Bass model. Specifically, in the formation of market potential, we consider the exponential growth of new energy vehicle market potential caused by improvements in product value. In the formation process of the already purchased group, we consider that new energy passenger vehicle diffusion depends on product value, which is related to the diffusion rate. Moreover, seasonal fluctuations are taken into account to make IBMPV more accurate in forecasting new energy passenger vehicle sales. We use quarterly data on new energy passenger vehicles in China from 2014 to 2023 to verify IBMPV, and compare its predictive performance with the Bass, Gompertz, Logistic and ARMAX models. The experimental results indicate that IBMPV achieves higher prediction accuracy than the above comparison models, highlighting the significance of product value in new energy passenger vehicle diffusion. This study presents a novel sales forecasting approach by integrating product value into the Bass model, providing a more accurate depiction of the diffusion mechanism of new energy passenger vehicles.

This study makes three key theoretical contributions. First, we improve the Bass model by incorporating product value, which can more accurately forecast new energy passenger vehicle sales and provide better explanations for the prediction results. By accounting for the impact of product value on consumer purchase intention and diffusion rate, we extend the Bass model, contributing to its further development. Second, this study provides empirical and detailed evidence of the relationship between product value and new energy passenger vehicle diffusion by incorporating product value into the Bass model, which has been indirectly confirmed in the field of consumer research [15,17,42]. We propose a product value function for new energy passenger vehicles grounded in value engineering theory, which not only synthesizes key product attributes influencing consumer purchasing decisions but also reflects technological progress in function and price, and then improves the Bass model based on product value, quantitatively estimating the influence of product value on new energy passenger vehicle diffusion. Therefore, our study is a combination of product value and the Bass diffusion model, extending research on product value to the product diffusion stage and revealing the diffusion mechanism of new energy passenger vehicles from the perspective of product value. Third, we find that technological innovation has a significant impact on new energy passenger vehicle diffusion, accelerating the diffusion process by influencing both market potential and the diffusion rate through product value. The market potential grows exponentially with product value, while the diffusion rate is mainly driven by product value and internal influences such as word-of-mouth. As product value increases, the external influence coefficient rises, whereas the internal influence coefficient declines. These insights are valuable for understanding the diffusion dynamics of new energy passenger vehicles and advancing research on sales forecasting.

Our study is beneficial for both new energy vehicle enterprises and policy makers. To promote the successful adoption of new energy passenger vehicles, both parties need to balance improvements in mainly two aspects: innovation and marketing. They can enhance product value through innovation, while optimizing marketing strategies to increase the attractiveness of innovative products. The improved model can provide decision support for new energy vehicle enterprises. For example, when making investment decisions, enterprises can allocate more resources to R&D to advance key technologies such as battery endurance, power performance, and fast charging capabilities. When conducting promotional marketing, they should not only strengthen the publicity of information on innovative attributes, but also promote experience activities such as test-driving to enhance consumers’ awareness and acceptance of new energy passenger vehicles. To further encourage adoption by imitators, enterprises can motivate adopters to share their experiences by improving customer service and creating online communities to amplify word-of-mouth effects. The model also offers guidance for policymakers. For example, the government can further bolster the development and strategic placement of infrastructure, while enhancing the promotion and dissemination regarding the attributes of new energy vehicles, thereby improving consumer perception of the product value of new energy passenger vehicles.

## Supporting information

S1 Data (RAR)
